# Heterogeneity reduces sensitivity of cell death for TNF-Stimuli

**DOI:** 10.1186/1752-0509-5-204

**Published:** 2011-12-28

**Authors:** Monica Schliemann, Eric Bullinger, Steffen Borchers, Frank Allgöwer, Rolf Findeisen, Peter Scheurich

**Affiliations:** 1 Institute for Automation Engineering, Laboratory for Systems Theory and Automatic Control, Otto-von-Guericke University Magdeburg, Germany; 2 Department of Electrical Engineering and Computer Science, Institut Montefiore, Université de Liège, Belgium; 3 Institute for Systems Theory and Automatic Control, University of Stuttgart, Germany; 4 Institute of Cell Biology and Immunology, University of Stuttgart, Germany

## Abstract

**Background:**

Apoptosis is a form of programmed cell death essential for the maintenance of homeostasis and the removal of potentially damaged cells in multicellular organisms. By binding its cognate membrane receptor, TNF receptor type 1 (TNF-R1), the proinflammatory cytokine Tumor Necrosis Factor (TNF) activates pro-apoptotic signaling via caspase activation, but at the same time also stimulates nuclear factor κB (NF-κB)-mediated survival pathways. Differential dose-response relationships of these two major TNF signaling pathways have been described experimentally and using mathematical modeling. However, the quantitative analysis of the complex interplay between pro- and anti-apoptotic signaling pathways is an open question as it is challenging for several reasons: the overall signaling network is complex, various time scales are present, and cells respond quantitatively and qualitatively in a heterogeneous manner.

**Results:**

This study analyzes the complex interplay of the crosstalk of TNF-R1 induced pro- and anti-apoptotic signaling pathways based on an experimentally validated mathematical model. The mathematical model describes the temporal responses on both the single cell level as well as the level of a heterogeneous cell population, as observed in the respective quantitative experiments using TNF-R1 stimuli of different strengths and durations. Global sensitivity of the heterogeneous population was quantified by measuring the average gradient of time of death versus each population parameter. This global sensitivity analysis uncovers the concentrations of Caspase-8 and Caspase-3, and their respective inhibitors BAR and XIAP, as key elements for deciding the cell's fate. A simulated knockout of the NF-κB-mediated anti-apoptotic signaling reveals the importance of this pathway for delaying the time of death, reducing the death rate in the case of pulse stimulation and significantly increasing cell-to-cell variability.

**Conclusions:**

Cell ensemble modeling of a heterogeneous cell population including a global sensitivity analysis presented here allowed us to illuminate the role of the different elements and parameters on apoptotic signaling. The receptors serve to transmit the external stimulus; procaspases and their inhibitors control the switching from life to death, while NF-κB enhances the heterogeneity of the cell population. The global sensitivity analysis of the cell population model further revealed an unexpected impact of heterogeneity, i.e. the reduction of parametric sensitivity.

## Background

Apoptosis is a cellular program essential for maintaining homeostasis in multicellular organisms. It represents the most common form of physiological cell death [1-4]. Tight control of apoptotic signaling is essential, as its downregulation may lead to cancer or autoimmune diseases [5,6]. Contrarily, atrophy, as in ischemic damage or neurodegenerative disorders such as Alzheimer's, Huntington's, and Parkinson's diseases, is characterized by excessive apoptotic activity [7-9]. Apoptosis can be induced by intrinsic and extrinsic signaling pathways that are highly regulated and interconnected and can be counteracted by anti-apoptotic signal pathways [10]. Thus, obtaining insights into the complex regulatory network related to apoptosis is important, yet challenging. Here we use a global sensitivity analysis on an experimentally validated cell ensemble model to illuminate apoptosis signaling.

An important extrinsic mediator of apoptosis is the cytokine Tumor Necrosis Factor (TNF). TNF exhibits potent antitumoral activity, but also represents a major activator of innate immune responses with strong pro-inflammatory and pathophysiological activities [11]. Soluble TNF interacts with and activates the membrane receptors TNF receptor type 1 (TNF-R1, CD120a, p55/60), whose activation triggers different cellular responses, including two seemingly contradictory ones. On the one hand, TNF-R1 induces cell proliferation by activating the transcription factor NF-κB [12]. On the other hand, internalized TNF-R1 initiates programmed cell death through the activation of initiator caspases [13,14]. There exists a second cell membrane receptor specific for TNF, called TNF-R2 (CD120b, p75/80), but this receptor can be activated only by the membrane integrated preform of TNF [15] and is not taken into account here.

The present study was performed with KYM-1 cells, a human rhabdomyosarcoma derived cell line, not affected in TNF sensitivity by overexpression of either bcl-2 or bcl-xl [16]. Therefore, apoptosis occurs independently of the mitochondrial pathway, and thus KYM-1 represents a model for TNF-induced apoptosis in so-called type 1 cells [17]. Another advantageous property of KYM-1 cells, in contrast to most other cell lines, is their high sensitivity to the apoptotic effects of TNF, even in the absence of a metabolic or DNA synthesis inhibitor [18]. As a consequence, apoptosis develops rapidly in these cells.

**Figure 1:**
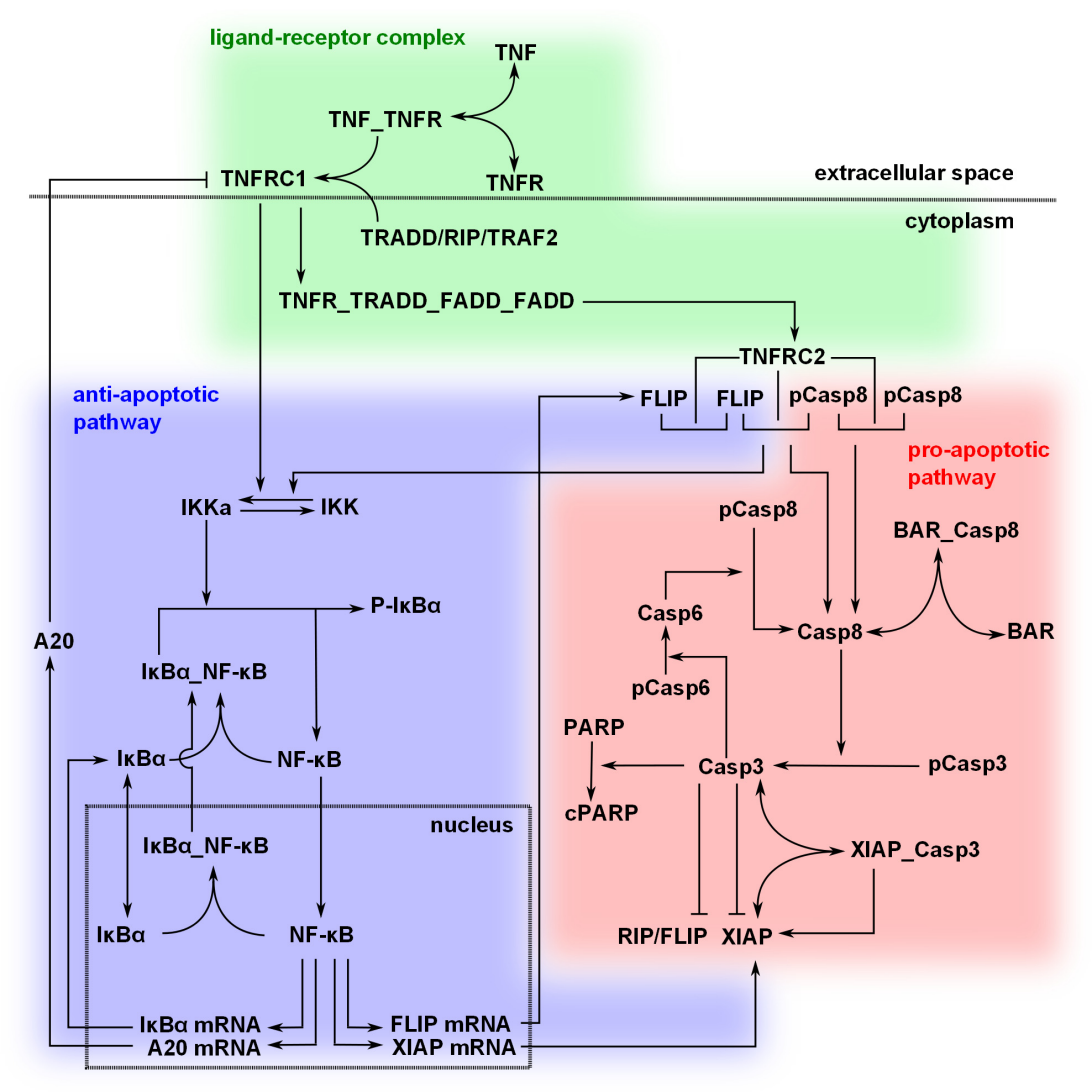
**Illustration of the Signaling Pathways and Interactions Initiated by TNF-R1**. Ligand stimulated TNF-R1 activates two major signaling pathways, pro- and anti-apoptotic cellular responses (red and blue background, respectively). The ligand-receptor interaction and signal complex formation is outlined in the green area.

The reaction network considered here is schematically depicted in Figure 1 and consists of three modules: the TNF-R1 receptor signaling complex (green background), the NF-κB pathway (blue background) and the caspase activation pathway (red background). The pro- and anti-apoptotic TNF-R1 signaling pathways are internally and mutually regulated via feedback loops leading to a highly complex and dynamic behavior. Obviously, the fine-tuning of the intracellular signaling pathways' regulatory circuits dictates the resulting cellular response (cell death or survival) in the end.

A key feature of the apoptotic program is its switch-like behavior, where the transition from life to death is rapid and not synchronized within a population of clonal cells [19]. Cell-to-cell variability is a hallmark of many biological phenomena, not only in induction of apoptosis [20], but also in adaptivity [21], immune response [22], stress response [23], differentiation and development [24] and cancer cell drug resistance [25]. Cell-to-cell variability can lead to different qualitative behavior at the single cell level but also in cell populations, in particular in systems showing bistability (all-or-nothing effects) or oscillatory behavior [26,27]. Causes for the intercellular heterogeneity can be stochastic fluctuations or deterministic, such as genetic/epigenetic differences or differential cell cycle phases. For human cells, protein levels of most signaling networks remain constant over several days [28] as they are tightly regulated [29]. Therefore, it seems reasonable to assume that life-death decisions of individual cells are executed on a deterministic basis.

Investigation of the complex, dynamic and highly regulated integration of TNF-induced pro and anti-apoptotic signals is facilitated by mathematical modeling based on the combination of quantitative and dynamic experimental data with mathematical modeling [30]. This abstraction helps testing hypotheses and provides insight into the governing mechanisms and their quantitative and dynamic features [31], which can be particularly intricate in apoptosis signaling [32]. Also see [33] for a review of apoptosis modeling.

A modeling framework for cell-to-cell variability is the cell ensemble modeling approach. This combines a large number of individual (cell) models that differ in some key parameters. This approach is particularly suitable for the description of unsynchronized cell cultures as it allows for the reproduction of the behavior of both single cells as well as whole cell populations. Successful application examples in the literature focus on heterogeneous cultures of *E. coli *growth [34], yeast metabolic oscillations [35] as well as TRAIL-induced apoptosis [20].

This study analyzes the competing pro and anti-apoptotic TNF signaling pathways with their crosstalk mechanisms within a heterogeneous cell population. The experimentally validated cell population model enables us to analyze the dynamics of TNF-R1 responses in more detail than would be possible by experiments alone. Results from this study underline the importance of the stimulus duration for the resulting cellular phenotype and the particular importance of the receptor concentration for the time of death development, as well as of NF-κB for cell-to-cell heterogeneity regarding the time of death. The analysis required defining a novel global sensitivity measure for heterogeneous populations: the linearized dependency of the time of death versus each population parameter. The sensitivity analysis of the cell population revealed that the nominal cell is much more sensitive than the cell population. The performed model analyses highlight the necessity, for a heterogeneous population, of quantifying the sensitivity on the population level, and not just of the nominal cell, even though this is much more convenient, computationally speaking.

## Results

The result section is structured as follows. First, experimental data on the time and dose dependency of TNF-R1-mediated signaling are shown. This led to the development of a cell ensemble model. Then, we present a model-based sensitivity analysis of the time of death, before concluding with an analysis of the NF-κB module on the heterogeneity within the population using *in-silico *knockout cells.

### Time and Dose Dependency of TNF-R1-mediated Cellular Responses

**Figure 2:**
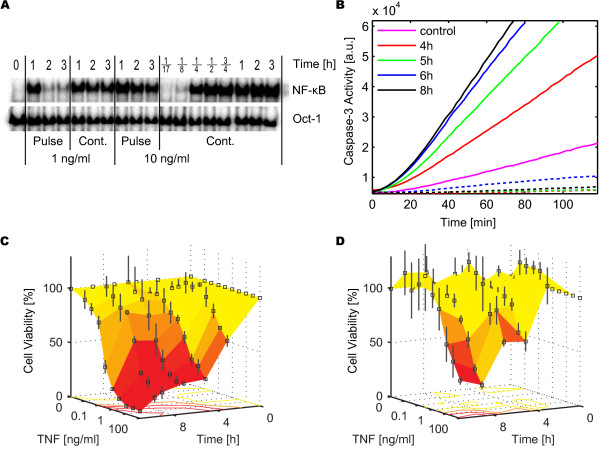
**Experimentally obtained Dynamic Dose-Responses**. (A) NF-κB in the nucleus after 1 ng/ml and 10 ng/ml TNF for 30 minute pulse or continuous stimulation, as shown by EMSA at different time points. Oct-1 served as loading control. (B) Caspase-3 activity assay for continuous stimulation of 1 ng/ml TNF (solid lines) measured 4-8 h post stimulus. Control experiments without TNF (magenta) and with the caspase inhibitor Z-VAD-fmk (dashed lines). (C) and (D) Cell viability over time for 10 different TNF concentrations (0-100 ng/ml TNF). The surfaces connect the measured data points. Continuous (C) and 30 minute pulse (D) stimulations, both including error bars from three independent experiments. The isoclines (lines connecting points of same cell viability) are shown projected onto the 0% planes.

Two important outputs of TNF-R1 signaling are the concentration of nuclear NF-κB and Caspase-3 activity. As NF-κB induces the expression of the caspase inhibitors XIAP and FLIP, it serves as a marker of the anti-apoptotic cell response. The experimental data on the concentration of active nuclear NF-κB indicated a rapid activation after stimulation with 10 ng/ml TNF (Figure 2A). For pulse stimulations, the NF-κB activity decreased after 1 hour, while it remained at a high level for continuous stimulations. The experiment shown in Figure 2A is representative for 11 different experiments, where cells had been stimulated with 0.3, 1 or 10 ng/ml TNF given either as a 30 minute pulse or continuously. These experiments consisted of 12-18 measurements each, with a total of 186 data points.

Caspase-3 activity increased over a time window of several hours (Figure 2B). Similarly, the quantification of cell viability over time revealed that the apoptotic response developed significantly later, occurring no earlier than 3 hours post stimulus (Figure 2C and 2D). A sigmoid correlation between TNF stimulus strength and viability could be observed: higher TNF concentrations resulted in earlier cell death and higher cell death rates (Figure 2C and 2D, see also Additional File 1 showing the same data in 2D panels). Differences between pulse and continuous TNF stimulation were most visible at intermediate concentrations (1-10 ng/ml TNF), where a TNF pulse did not induce significant apoptosis, whereas continuous stimulation did (Figure 2C). However, even for nearly saturating TNF concentrations (≥ 30 ng/ml), continuous TNF stimulation led to a faster response and higher death rate as compared to a respective TNF pulse (Figure 2D). For very low TNF concentrations (≤ 0.1 ng/ml), neither a continuous nor a pulse TNF treatment induced significant apoptosis.

The sigmoidal dose response and the different time scales highlighted the complexity of the TNF-induced signaling. To gain further insight into the regulatory mechanisms of this system, we first developed a mechanistic single cell model of TNF induced pro and anti-apoptotic signaling describing the signal transduction within a median cell. The model structure corresponds to the pathway shown in Figure 1. See Additional File 2 for a detailed description of the model and Additional File 3 for its SBML code.

**Figure 3:**
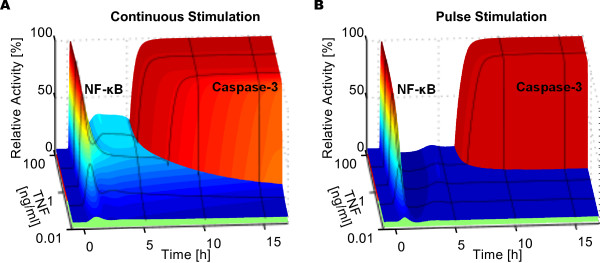
**Simulations of the Single Cell Model**. (A) and (B) Single cell time course simulations of relative NF-κB translocation into the nucleus and Caspase-3 activation for TNF concentrations in the range of 0.01 - 100 ng/ml; (A) for continuous TNF stimulation and (B) for 30 minute TNF pulse stimulation. Relative activities represent the concentration time courses divided by their maximum values. For NF-κB, the relative activity is color coded such that blue depicts a low, yellow a medium and red a high nuclear accumulation. For Caspase-3, the color of a complete trajectory stands for the time of death: early death in red, late death in yellow, cell survival in green.

To illustrate the model behavior, we performed time course simulations of the nominal cell model over 16 hours post stimulus with TNF concentrations ranging from 0.01 to 100 ng/ml, both for continuous (Figure 3A) and 30 minute pulse stimulations (Figure 3B). NF-κB was rapidly activated even at low TNF concentrations, and converged to a higher activation level in the case of a continuous TNF stimulation (Figure 3A versus Figure 3B). For both types of stimuli, Caspase-3 activation occurred after a delay of a few hours with a switching duration of only a few minutes. TNF pulse stimulations resulted in later responses of Caspase-3 activation, and no activation at all was observed for the lowest concentration range. Interestingly, a higher TNF stimulus led to a higher and an earlier NF-κB translocation as well as earlier Caspase-3 activation.

The experimental data presented in Figure 2 were based on averaging measurements of a population of cells. To reproduce this data, we established a mathematical model of a cell population in the form of a cell ensemble model, in which all production rates were lognormally distributed within the cell population, see Additional File 2. For a specific stimulus, the qualitative response varied between individual cells of the cell population: some cells survived, some died with a time of death from 2 hours post stimulus onwards, where cell death is defined as the time point of 50% cleavage of the Caspase-3 substrate PARP. The cell ensemble model was validated using cytotoxicity assays at various concentrations of TNF as well as using Western blotting, caspase activity assays and microinjection experiments, see Additional File 2.

### Sensitivity of the Time of Death

**Figure 4:**
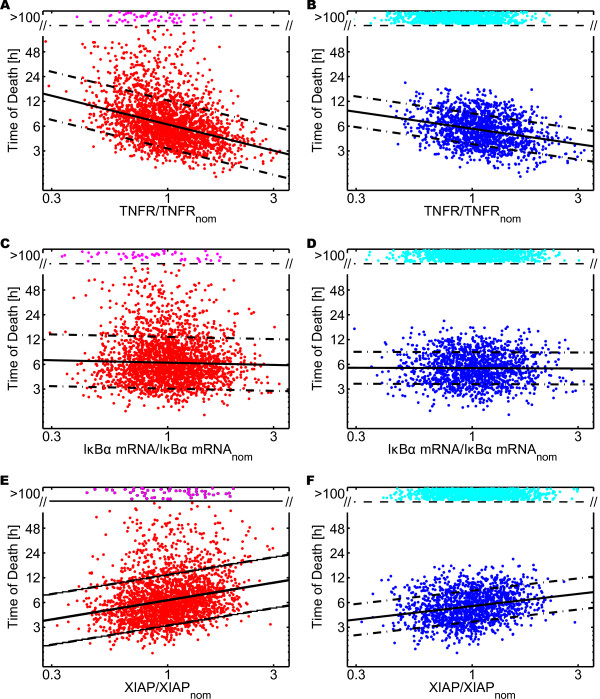
**Cell Population Sensitivity Analysis**. (A) - (F) Time of death for 2500 cells with lognormally distributed production rates, plotted as a projection versus one of these rates normalized by its nominal value. Cells not dying within 100 hours post stimulus with TNF are classified as survivors and plotted as magenta or cyan dot in the zone "> 100" at a random ordinate, the others are plotted as red or blue dot. The number of dying cells is approximated using robust linear regression (black solid lines) and an estimate of the standard deviation of the error (black dash-dotted lines). (A), (C), (E) continuous stimulation with 10 ng/ml TNF. (B), (D), (F) 30 minute pulse stimulation with 10 ng/ml TNF. (A) - (B) projection with respect to the production rate of TNF receptors, (C) - (D) to IκBα mRNA expression and (E) - (F) to XIAP production.

To gain a deeper understanding of the importance of the different parameters, we studied the sensitivity of the time of death with respect to the population parameters. As a global sensitivity measure, we defined the slope of the linear approximation of time of death versus each population parameter on a log-log plot. Figure 4 depicts the projection of the time of death of individual cells with respect to three of the population parameters (production rate of TNF receptor, IκBα mRNA expression and XIAP production). The linear approximation obtained using robust linear regression is shown as solid black lines. Its slope is our global sensitivity measure. As TNFR had a negative slope, the more TNFR was produced the earlier the cells died. XIAP, on the other hand, had a positive slope, thus more XIAP delayed cell death. For IκBα, no clear trend was visible.

**Figure 5:**
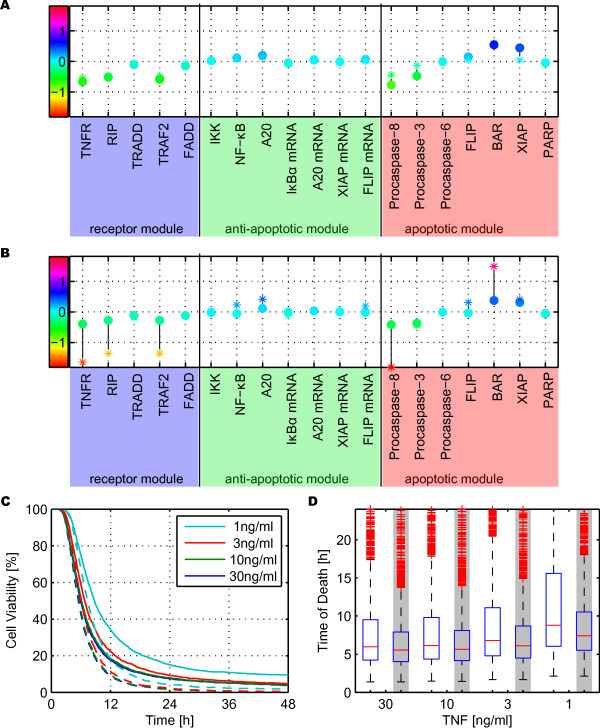
**Sensitivity and Heterogeneity Analysis of Wild Type and NF-κB Knockout Model**. (A) - (B) Global sensitivity measures defined as the slope of the linear correlation of time of death versus each population parameters, obtained using robust linear regression on a log-log plot. Dots: cell population sensitivity, stars: sensitivity of the nominal cell. The color coding highlights the slope value: the more red the higher the magnitude of the sensitivity, and the vertical black lines connect cell population and nominal cell sensitivities of the same parameter. (A) continuous stimulation, (B) 30 minutes pulse stimulation. (C) - (D) Cell viability as a function of time for different continuous stimulation of TNF concentrations (1, 3, 10, 30 ng/ml). (C) Solid lines: wild type cell population, dashed lines: without activation of the NF-κB-pathway. (D) Box plots of the distribution of the time of death within the cell population for different TNF concentrations. The solid red line depicts the median values of the cell populations; blue box: second and third quartiles; dashed line: whiskers with a maximum length of 1.5 interquartile range; red pluses: outliers. White background: wild type cells, grey background: cells without NF-κB activation.

Figure 5 shows the value of the global sensitivity measure for each one of the 19 distributed parameters, in a population of 2500 cells stimulated *in silico *with 10 ng/ml TNF, either continuously (Figure 5A) or applied as a 30 minute pulse (Figure 5B). In both cases, the sensitivity of the cell population was compared to the sensitivity analysis performed on the nominal cell using a multiplicative perturbation of 1.1, increasing and decreasing each parameter. While in many cases the results of nominal and cell population sensitivity were similar, there were significant differences, particularly for pulse stimulations. There, several nominal sensitivities were significantly higher than the corresponding sensitivity of the cell population, see especially TNFR, RIP, TRAF2, Procaspase-8 and BAR.

Analyzing the cell population sensitivity revealed that the parameters can be divided into three groups. In the first one, an increase of the parameter resulted in later death. Members of this group are the caspase inhibitors XIAP and BAR. Members of the second group caused the opposite effect: a larger parameter value correlated with earlier death. This group includes the TNF receptor and its adaptor proteins RIP, TRAF2, as well as the Procaspases-3 and -8. The remaining parameters had little impact on the time of death. Most parameters exhibited similar sensitivities for continuous and pulse stimulation, even though cell death was delayed in the nominal case by approximatively 3 hours for a 30 minute pulse compared to the respective continuous stimulation. Different sensitivities for pulse and continuous stimulation were only visible for TNFR, RIP, TRAF2 A20, BAR and XIAP. For all of them, the magnitude of the sensitivity was smaller for pulse stimulation compared to continuous stimulation.

### NF-κB Module Enhances Heterogeneity

The sensitivity analysis suggested that the NF-κB module was irrelevant for the timing of death, see Figure 4C and 4D. To confirm or refute this hypothesis, we studied the importance of the NF-κB pathway by comparing "wild type" cells to cells manipulated *in silico *to show no TNF induced NF-κB activation. Mathematically, NF-κB knockout cells were modeled by setting the rate of the irreversible reaction *release and degradation of bound IκBα *IKKa + IκBα:NF-κB → IKKa + NF-κB + PIκBα to zero.

For continuous stimulation, a higher dose led to earlier death and the NF-κB knockout cells died earlier than the wild type ones, especially for low dosage concentrations (Figure 5C). The reduced standard deviation, in particular, revealed much more homogeneous behavior for the knockout cells than for the wild type cells (Figure 5D and Table 1). For pulse stimulation, the survival rate was significantly dosage dependent (data not shown) and wild type cells were more protected from death (Table 1).

**Table 1:** Impact of the NF-κB Pathway

	TNF	30 ng/ml	10 ng/ml	3 ng/ml	1 ng/ml
Continuous	Death rate	+1.46%	+1.59%	+2.93%	+8.15%
	
	Median time of death	-6.25%	-6.85%	-8.55%	-10.6%
	
	Relative std	-57.9%	-53.3%	-34.1%	-18.3%

Pulse	Death rate	+11.7%	+13.5%	+19.0%	+35.2%
	
	Median time of death	+0.99%	+0.37%	+3.27%	+3.52%
	
	Relative std	+3.84%	+8.91%	-2.72%	-0.80%

Comparison of NF-κB knockout cells and wild type cells for different stimulations (continuous and 30 minute pulse of TNF at concentrations of 1, 3, 10 or 30 ng/ml). Death rate, median time of death and standard deviation normalized by the median (relative std) are shown here for the knockout cells normalized by the corresponding wild type cell value.

## Discussion

The dose-response relationship of TNF induced signaling follows an intricate pattern. Low TNF concentrations barely induce any system response. For high TNF concentrations, the system is insensitive to stimulus changes due to receptor saturation. In between, especially in the range of 1-10 ng/ml TNF, small stimulus changes can lead to different phenotypes, or at least have a significant impact on timing and amplitude of the signaling processes. The precise knowledge of this window of high cellular sensitivity to changes in stimulus strength is of particular interest for the development of therapeutic strategies.

Heterogeneity within a clonal cell population is an essential property of many biological signaling mechanisms, as is also the case for apoptosis signaling. After an extracellular stimulus, the final decision of cellular death versus survival deterministically depends on each particular cell's epigenetic state. While some cells quickly undergo apoptosis, others only slowly develop the final destructive program and therefore the time of death within the whole population is spread over many hours. One particular group of cells even survives the stimulus that is lethal to other cells. This corresponds to the observed partial TNF resistance, where the level of resistance is dependent on time and stimulus dosage. This observed cell to cell heterogeneity can well be described by cell ensemble models as demonstrated with the proposed model as well as by others.

Global sensitivity analysis revealed that out of all protein production rates those of BAR and XIAP had, by far, the highest positive correlation with the time of death, i.e. overexpressing these caspase inhibitors delayed cell death significantly. Procaspase-8, TNF-Receptor, TRAF2, RIP and Procaspase-3 were the most sensitive parameters showing a negative correlation, thus their increase advances cell death.

A comparison of the sensitivities of the cell population with the nominal cell reveals a much lower sensitivity to parametric variations of the cell population. The sensitivity is also enhanced in NF-κB knockout cells even though the model shows that the time of death is insensitive to the parameters of the NF-κB module. Thus, our analysis reveals a hidden advantage of heterogeneous populations resulting in a lower sensitivity to parameter variations.

## Conclusion

This paper highlights the complexity of analyzing the sensitivity of cell population models. The proposed global sensitivity analysis method allowed for a quantification of the importance of the population parameters within the heterogeneous cell population. The analysis results were dependent on the input signal, i.e. its intensity and duration. The heterogeneity within a cell population was shown to reduce the sensitivity as determined by a global sensitivity analysis of the time of death. Even though the NF-κB module seemed unimportant from a sensitivity analysis point of view, it not only increases the expression of anti-apoptotic proteins, but also enhances the heterogeneity.

In summary, the higher computational effort of calculating the sensitivity for a cell population, as opposed to a single cell, is necessary in order to understand the importance of the different parameters.

## Methods

### Cell Culture, Stimulation and Plasmids

The human rhabdomyosarcoma cell line KYM-1 was kindly provided by Dr. M. Sekiguchi, University of Tokyo, Japan [36]. Cells were maintained in RPMI 1640 supplemented with L-Glutamine and 10% heat inactivated fetal calf serum (FCS) and splitted every 2-3 days. All experiments were carried out using the TNF-R1 selective TNF mutant CysHisR32W/S86T-TNF, here referred to as TNF [37-39]. For pulse stimulations, TNF was removed after 30 minutes by three washes with RPMI medium. Subsequently, fresh culture medium was added. Controls revealed that this washing procedure was highly effective to remove all free TNF. For continuous stimulations, no fresh TNF and culture medium were added. The plasmid pEGFP-p65 was kindly provided by Dr. J. A. Schmid, University of Vienna [40].

### Preparation of Nuclear Protein Extracts

10^6 ^KYM-1 cells were seeded in 6-well plates, cultivated over night and stimulated the following day as mentioned for each experiment. Nuclear protein extracts were obtained by lysing the cells [41]. Total protein concentrations were determined with the Bradford protein assay reagent (Protein Assay, BioRad, Hercules, USA) and photometric extinction measurement at 595 nm. For all samples, identical protein amounts were employed.

### Electrophoretic Mobility Shift Assays (EMSAs)

Nuclear protein extracts were subjected to EMSAs with [γ-^32^P]-ATP end-labeled oligonucleotides with one NF-κB binding site (5'-AGTTGAGGGGACTTTCCCAGG-3'). Ten micrograms of each probe were incubated with 4 μg poly dI-dC (GE Healthcare, Uppsala, Sweden) for 15 minutes at room temperature and thereafter with radioactively labeled oligonucleotides for 10 minutes. After addition of the DNA sample buffer, EMSA was performed in a 6% polyacrylamide gel for 90 minutes at 22 mA. Vacuum dried gels were exposed to an X-ray screen over night, the autoradiographs were scanned (Molecular Probes Storm 860 Phosphoreader, Invitrogen, Carlsbad, USA). Oct-1 served as a loading control.

### Caspase Activity Assays

KYM-1 cells (5·10^5^/well) were seeded and cultivated overnight in 6-well plates. After 4, 5, 6 or 8 hours incubation time with 1 ng/ml TNF, cytoplasmatic cell lysates were obtained. As control experiments, the same stimulation was performed on cells preincubated with 20 μM Z-VAD-fmk. On ice, 15 μg total protein in a volume of 20 μl were added to 125 μl caspase activity buffer, containing the protease inhibitor Complete (1×) and the reducing agent DTT (1 μM final concentration) and 1 μl Caspase-3 substrate AC-DEVD-AMC. Samples were measured by using an automated fluorimeter (Tecan Infinite M200) at 37°C for 2 hours, in which emission was measured in 2 minute intervals, at wavelengths of 380 nm (excitation) and 460 nm (emission). Caspase amount was quantified as the median value of each slope.

### Cytotoxicity Assays

KYM-1 cells (10^4^/well) were seeded and cultivated overnight in 96-well microtiter plates (Greiner Bio-One GmbH, Frickenhausen, Germany). The next day, cells were treated with serial dilutions of TNF, ranging from 100 to 0.01 ng/ml for several periods of time. Supernatants were discarded, cells were washed with PBS and cell viability was determined using crystal violet (CV) staining [42]. Background was defined using CV only and was subtracted from the signal. Values were normalized with respect to untreated controls.

### Computational Tools

The nominal, single cell model is available in Systems Biology Markup Language (SBML), see Additional File 3. Simulations were performed with Matlab Version 7.1.0.246 (R14) Service Pack 3 (Mathworks) with the built-in variable order, multistep solver *ode15s*, which is based on numerical differentiation formulas, as the differential equations are stiff.

The cell ensemble model was obtained by varying all expression rates independently from each other, lognormally distributed with a standard deviation σ = 0.148 and as mean the value of the single cell model. For each sample of the population, the initial condition was the corresponding steady state that has no activated caspases.

Robust linear regression and the estimate of the standard deviation of the error were calculated using the Matlab commands robustfit from the Statistics Toolbox and polyval, respectively.

Direct comparison of simulation with experimental data required their normalization. First, the background intensity of each measurement was determined and subtracted from the data. Measurements with signal intensities lower than the background were set to zero. The values were then normalized by the maximum of the time course to obtain relative intensities in the range of 0 to 100%.

## Authors' contributions

MS conceived and carried out all wet-lab experiments as well as the modeling. PS participated in the design of the experimental study. MS and RF conceived the theoretical study. SB collaborated on the cell ensemble modeling. EB performed the model analyses. FA participated in coordinating the study. MS, EB and PS wrote the manuscript. All authors read and approved the final manuscript.

## Supplementary Material

Additional file 1 **Cytotoxicity Assays**. Quantification of cell viability of KYM-1 cells for different TNF concentrations (0-100 ng/ml TNF). The experiments were performed at 5 different times post stimulus (color coded), for continuous (solid lines) and 30 minute pulse (dashed lines) stimulations. Both included error bars from three independent experiments. Figures 2C-D show this data in a 3-D view. Click here for file

Additional file 2 **Detailed Description of the Mathematical Model and Model Validation**. Detailed description of the mathematical model including experimental data for model validation. Click here for file

Additional file 3 **SBML Code of the Nominal Cell Model**. SMBL code of the nominal cell model. Also available in the BioModels data base as MODEL1112210000. See http://sbml.org/ for software to simulate or visualize SBML models. Click here for file
