# Supplementary material for: Heterogeneity reduces sensitivity of cell death for TNF-Stimuli
Source: BMC Syst Biol. 2011 Dec 28;5:204. doi: 10.1186/1752-0509-5-204 (PMC3313907; doi:10.1186/1752-0509-5-204)
Supplement: Additional file 2 — Detailed Description of the Mathematical Model and Model Validation. Detailed description of the mathematical model including experimental data for model validation. [file 1752-0509-5-204-S2.PDF]

Supporting Information for Schliemann et al., 2011

This supplement gives further information on the proposed mathematical model of TNF induced pro- and anti-apoptotic signaling. First, the mathematical model of a nominal cell is described, with a particular emphasis on the parameter estimation involved in its development. The model is fully described in Tables S2.1 – S2.3, which list species, reactions and compartments of the model. The second part defines the cell ensemble model. Finally, the cell ensemble model is validated with different experimental data sets.

Single Cell Mathematical Model

The mathematical model of TNF-R1 induced pro- and anti-apoptotic signaling is based on a combination of published models [1-2] that have been significantly expanded by novel parts describing the signal complex formation, the NF-κB activation mechanism, and the crosstalk of anti- and pro-apoptotic pathways. See Figure 1 in the main paper.

The model consists of 47 compounds and 88 reactions, including mRNA synthesis, translation and transportation between extracellular space, cytoplasm and nucleus, as well as formation and dissociation of complexes. See Table S1 for a list of species and Table S2 for the list of reactions. All occurring reactions were modeled according to the law of mass action. As 18 reactions are reversible, the model contains 106 kinetic parameters.

The model parameters were adjusted to KYM-1 cells. Some of the parameters could be determined from our own experimental data (Figure S 2.1). From Figure 2C - D, the following values were estimated as time of death of a nominal cell: 5.25h for 10 ng/ml and 7.5h for 1 ng/ml continuous TNF stimulation; for 30 minute pulse stimulation, 7.0h for 10 ng/ml and survival for 1 ng/ml.

Most other parameters were taken from literature [1-4]. The remaining parameters were adjusted to published experimental data, e.g. reproducing constitutive NF-κB shuttling with 9% of the total NF-κB and 17% of the total IκBα already present in the nucleus without TNF stimulation [5], or to binding studies of TNF and TNF-R1 [6], using Monte-Carlo based optimizations.

The assumed extracellular volume of 420 μm^3 corresponded to the amount of culture medium available per cell in our standardized cytotoxicity assay. Values of a representative cell close to the median volumes were estimated using 3D-microscopy (data not shown). The volumes of the cellular compartments were chosen as 3.2 μm^3 for the cytoplasm and 1/3 of that for the nucleus. The compartment volumes are shown in Table S2.3.

The initial conditions for the model were chosen such that all states were in the equilibrium without TNF or activated caspases (the life steady state). Due to the permanent nuclear shuttling of NF-κB and IκBα, this steady state could only be calculated numerically.

The nominal, single cell model is available in Systems Biology Markup Language (SBML). See Supplementary material model_code.xml.

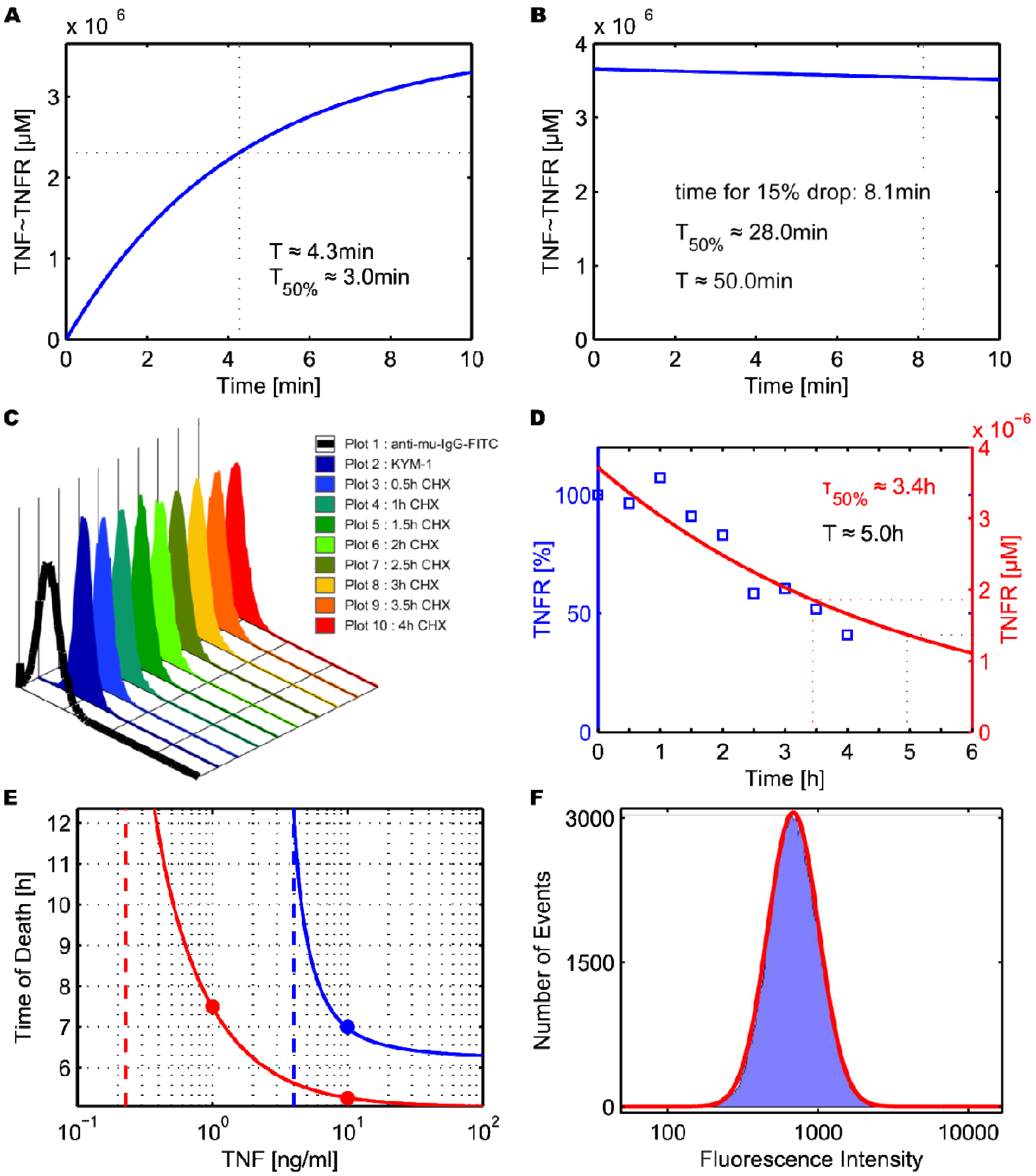

Figure S2.1. Comparison of Experiments and Simulation employed for the Model Calibration. (A) Binding of TNF to the TNF receptor, reaching 50% after 4.3 minutes. This is comparable to [6]. (B) Unbinding of TNF from the TNF receptor, with a decrease of 15% within approx. 8 minutes, as [6]. (C) Degradation rate of the TNF receptor: our own experimental data obtained by blocking protein production with CHX and quantifying the number of receptors using flow cytometry.(D) Degradation rate of the TNF receptor: our own experimental data as shown in(C) (blue squares, left axis), model simulation (red line, right axis). (E) Time of death for 1 and 10 ng/ml TNF of a median cell as estimated from cytotoxity assays, Figure S1: dots. Time of death for different TNF stimulation (solid lines) and

minimal lethal TNF concentration (dash-dotted line) as calculated from the mathematical model of a nominal cell. Red: continuous, blue: 30 minute pulse stimulation. (F) Cell surface TNF-R1 distribution, experimentally determined by FACS analysis (in blue) and approximated by a lognormal distribution (in red).

Table S2.1: List of Species

List of all species of the model, with their compartment, initial condition, database information (uniprot [7] or ensemble[8] IDs) and additional information. The numerical values are rounded to three significant digits.

	Name	Compartment	Initial	Database Information	Comment
1	TNFR_E	extracellular	3.72e-6 µM	uniprot:P19438	TNF receptor1 on KYM-1 as dimers, 3000 binding sites/cell [9]
2	TNF_E	extracellular	0.0002 µM	uniprot:P01375	Tumor Necrosis Factor; CysHisTNF R32W/S86T[10-13]
3	TNF:TNFR_E	extracellular	0 µM	uniprot:P19438|P01375	TNF-TNF-R1 complex \cite[6]
4	TNFR	cytoplasm	8.75e-5 µM	uniprot:P19438	freshly produced TNF receptor1
5	RIP	cytoplasm	0.0633 µM	uniprot:Q13546	Receptor-interacting protein
6	TRADD	cytoplasm	0.0917 µM	uniprot:Q15628	TNFR1-associated DEATH domain protein
7	TRAF2	cytoplasm	0.103 µM	uniprot:Q12933	TNF receptor-associated factor 2
8	FADD	cytoplasm	0.0967 µM	uniprot:Q13158	FAS-associated death domain protein
9	TNF:TNFR:TRADD	cytoplasm	0 µM	uniprot:P19438|P01375|Q15628	TNF~TNFR~TRADD complex
10	TNFRC1	cytoplasm	0 µM	uniprot:P19438|P01375|Q15628|Q129	TNF-R1-Complex1 includes: RIP and TRAF2

				33|Q13546	
11	TNFRCint1	cytoplasm	0 µM	uniprot:P19438|P01375|Q15628|Q12933|Q13546	transitional receptor stage1
12	TNFRCint2	cytoplasm	0 µM	uniprot:P19438|P01375|Q15628	transitional receptor stage2
13	TNFRCint3	cytoplasm	0 µM	uniprot:P19438|P01375|Q15628|Q13158	transitional receptor stage3
14	TNFRC2	cytoplasm	0 µM	uniprot:P19438|P01375|Q15628|Q13158	TNF-R1-Complex2 includes: FADD
15	FLIP	cytoplasm	0.00856 µM	uniprot:O15519	FLICE-inhibitory protein
16	TNFRC2:FLIP	cytoplasm	0 µM	uniprot:P19438|P01375|Q15628|Q13158|O15519	TNFRC2~FLIP complex
17	TNFRC2:pCasp8	cytoplasm	0 µM	uniprot:P19438|P01375|Q15628|Q13158|Q14790	TNFRC2~pCasp8 complex
18	TNFRC2:FLIP:FLIP	cytoplasm	0 µM	uniprot:P19438|P01375|Q15628|Q13158|O15519|O15519	TNFRC2~FLIP~FLIP complex

19	TNFRC2:pCasp8:pCasp8	cytoplasm	0 μM	uniprot:P19438|P01375|Q15628|Q13158|Q14790|Q14790	TNFRC2~pCasp8~pCasp8 complex
20	TNFRC2:FLIP:pCasp8	cytoplasm	0 μM	uniprot:P19438|P01375|Q15628|Q13158|O15519|Q14790	TNFRC2~FLIP~pCasp8 complex
21	TNFRC2:FLIP:pCasp8:RIP:TRAF2	cytoplasm	0 μM	uniprot:P19438|P01375|Q15628|Q13158|O15519|Q14790|Q13546|Q12933	TNFRC2~FLIP~pCasp8~RIP~TRAF2 complex
22	IKK	cytoplasm	0.2 μM	uniprot:O15111	Inhibitor-κB kinase in inactive form
23	IKKa	cytoplasm	0 μM	uniprot:O15111	active IKK
24	A20	cytoplasm	0.0326 μM	uniprot:P21580	A20-protein
25	NFkB	cytoplasm	3.61e-5 μM	uniprot:P19838	nuclear factor κB
26	IkBa	cytoplasm	0.000317 μM	uniprot:P25963	Inhibitor-κBα
27	IkBa:NFkB	cytoplasm	0.00472 μM	uniprot:P25963|P19838	IκBα~NF-κB complex
28	PIkBa	cytoplasm	0 μM	uniprot:P25963	P-IκBα

29	NFkB_N	nucleus	0.000655 μM	uniprot:P19838	nuclear NF-κB
30	IkBa_N	nucleus	0.00131 μM	uniprot:P25963	nuclear IκBα
31	IkBa:NFkB_N	nucleus	8.52e-5 μM	uniprot:P25963|P19838	nuclear IκBα~NF-κB complex
32	A20_mRNA	nucleus	5.27e-5 μM	ensembl:Homo_sapiens/Transcript/Summary?t=ENST00000237289	A20-mRNA; A20 gene: TNF α-induced protein3
33	IkBa_mRNA	nucleus	5.03e-5 μM	ensembl:Homo_sapiens/Transcript/Summary?t=ENST00000216797	IκBα-mRNA; IκBα gene: NF-κB inhibitor α
34	XIAP_mRNA	nucleus	0.000208 μM	ensembl:Homo_sapiens/Transcript/Summary?t=ENST00000371199	XIAP-mRNA; XIAP gene: baculoviral IAP repeat-containing 4
35	FLIP_mRNA	nucleus	0.000132 μM	ensembl:Homo_sapiens/Transcript/Summary?t=ENST00000309955	FLIP-mRNA; FLIP gene: CFLAR
36	BAR	cytoplasm	0.0886 μM	uniprot:Q9NZS9	Bifunctional apoptosis regulator

37	XIAP	cytoplasm	2.44 µM	uniprot:P98170	X-linked inhibitor of apoptosis protein
38	pCasp8	cytoplasm	1 µM	uniprot:Q14790	Procaspase-8
39	pCasp3	cytoplasm	0.25 µM	uniprot:P42574	Procaspase-3
40	pCasp6	cytoplasm	0.02 µM	uniprot:P55212	Procaspase-6
41	Casp8	cytoplasm	0 µM	uniprot:Q14790	active Caspase-8
42	Casp3	cytoplasm	0 µM	uniprot:P42574	active Caspase-3
43	Casp6	cytoplasm	0 µM	uniprot:P55212	active Caspase-6
44	BAR:Casp8	cytoplasm	0 µM	uniprot:Q9NZS9|Q14790	BAR~Caspase-8 complex
45	XIAP:Casp3	cytoplasm	0 µM	uniprot:P98170|P42574	XIAP~Caspase-3 complex
46	PARP	cytoplasm	0.521 µM	uniprot: P09874	Poly [ADP-ribose] polymerase 1
47	cPARP	cytoplasm	0 µM	uniprot: P09874	cleaved PARP

Table S2.2: List of Reactions

List of all reactions of the model, with substrate, reaction direction, product, reaction rate for forward (ka) and backward (kd) reaction, name and additional information. The numerical values are rounded to three significant digits.

	Substrate		Product	ka	kd	Name	Comment
1	TNFR	⟶	TNFR_E	0.001 1/s	---	TNFR transport into membrane	fast export of resynthesized TNF-Receptor
2	0	⟶	TNFR	8.75e-8 μM/s	---	TNFR production	[6, 14]; TNFR1 on KYM-1 as dimers (=3000 binding sites/cell); 3000(mo/cell)/6e5(μM/mo)/3.2e-12(cell volume)
3	TNFR_E	⟶	0	0.0235 1/s	---	TNFR degradation	TNFR1 on KYM-1 as dimers (=3000 binding sites/cell); degradation rate measured in own FACS experiment
4	0	⟷	RIP	6.33e-6 μM/s	0.0001 1/s	RIP turnover	[14]
5	0	⟷	TRADD	9.17e-6 μM/s	0.0001 1/s	TRADD turnover	[14]
6	0	⟷	TRAF2	1.03e-5 μM/s	0.0001 1/s	TRAF2 turnover	[14]
7	0	⟷	FADD	9.67e-6 μM/s	0.0001 1/s	FADD turnover	[14]
8	TNF:TNFR_E	⟶	0	0.0235 1/s	---	TNF~TNFR degradation	same as TNFR degradation
9	TNF:TNFR:TRADD	⟶	0	0.0235 1/s	---	TNF:TNFR:TRADD degradation	same as TNFR degradation
10	TNFRC1	⟶	0	5.6e-5	---	TNFR Complex1	same as TNFR degradation

				1/s		degradation	
11	TNFRC2	⟶	0	5.6e-5 1/s	---	TNFR Complex2 degradation	same as TNFR degradation
12	TNFRC2:FLIP	⟶	0	5.6e-5 1/s	---	TNFR Complex2~FLIP degradation	same as TNFR degradation
13	TNFRC2:FLIP:FLIP	⟶	0	5.6e-5 1/s	---	TNFR Complex2~FLIP~FLIP degradation	same as TNFR degradation
14	TNFRC2:pCasp8	⟶	0	5.6e-5 1/s	---	TNFR Complex2~Procaspase-8 degradation	same as TNFR degradation
15	TNFRC2:pCasp8:pCasp8	⟶	0	5.6e-5 1/s	---	TNFR Complex2~Procaspase-8~Procaspase-8 degradation	same as TNFR degradation
16	TNFRC2:FLIP:pCasp8	⟶	0	5.6e-5 1/s	---	TNFR Complex2~FLIP~Procaspase-8 degradation	same as TNFR degradation
17	TNFRC2:FLIP:pCasp8:RIP:TRAF2	⟶	0	5.6e-5 1/s	---	TNFR Complex2~FLIP~Procaspase-8~RIP~TRAF2 degradation	same as TNFR degradation
18	TNFR_E + TNF_E	⟷	TNF:TNFR_E	5.38e+3 1/μM/s	0.0277 1/s	TNF~TNFR binding and release	fitted [6]
19	TNF:TNFR_E + TRADD	⟶	TNF:TNFR:TRADD	5.75 1/μM/s	---	TNF~TNFR~TRADD building	fitted
20	RIP + TRAF2 + TNF:TNFR:TRADD	⟶	TNFRC1	1 1/s/(μM)^2	---	TNFR Complex1 building	RIP for NF-κB activation; RIP ubiquitination after TRAF2 [15]; TRADD-TRAF2-Complex [16]; fitted for an internalization T(50%) of 10-

							20min [6]
21	TNFRC1	⟶	TNFRCint1	0.00113 1/s	---	Receptor internalization step1	fitted to realize 45 min complex2-lag-phase [17]; internalization of TNFRC1
22	TNFRCint1	⟶	RIP + TRAF2 + TNFRCint2	0.00113 1/s	---	Receptor internalization step2	fitted to realize 45 min complex2-lag-phase[17]; dissociation of TRAF2 and RIP
23	2 FADD + TNFRCint2	⟶	TNFRCint3	0.121 1/s/(µM)^2	---	Receptor internalization step3	fitted to realize 45 min complex2-lag-phase[17]; recruiting of FADD
24	TNFRCint3	⟶	TNFRC2	0.114 1/s	---	Receptor internalization step4	fitted to realize 45 min complex2-lag-phase [17]; fusion with Golgi
25	TNFRC2 + FLIP	⟶	TNFRC2:FLIP	1 1/µM/s	---	FLIP recruitment to TNFR Complex2	DISC formation; [18]; fitted
26	FLIP + TNFRC2:FLIP	⟶	TNFRC2:FLIP:FLIP	1 1/µM/s	---	FLIP recruitment to TNFR Complex2~FLIP	[18]; fitted
27	TNFRC2 + pCasp8	⟶	TNFRC2:pCasp8	0.1 1/µM/s	---	Procaspase-8 recruitment to TNFR Complex2	Procaspase-8 at DISC; [18] [18-19]; fitted
28	TNFRC2:pCasp8 + pCasp8	⟶	TNFRC2:pCasp8:pCasp8	0.1 1/µM/s	---	Procaspase-8 recruitment to TNFR Complex2~Procaspase-8	Procaspase-8 homodimer at DISC; [18-19]; fitted
29	TNFRC2:pCasp8:pCasp8	⟶	TNFRC2 + Casp8	2.7 1/s	---	Caspase-8 activation by TNFR Complex2	Procaspase-8 homodimer at DISC leads to active Caspase-8; 2 pCasp8=1 Casp8; [18-19]; fitted
30	FLIP + TNFRC2:pCasp8	⟶	TNFRC2:FLIP:pCasp8	1 1/µM/s	---	FLIP recruitment to TNFR Complex2~Procaspase-8	[18-19]; fitted
31	TNFRC2:FLIP + pCasp8	⟶	TNFRC2:FLIP:pCasp8	1 1/µM/s	---	Procaspase-8 recruitment to TNFR Complex2~FLIP	[18]; fitted

32	TNFRC2:FLIP:pCasp8	⟶	TNFRC2 + Casp8	1.8 1/s	---	Caspase-8 activation by TNFR Complex2~FLIP~Procaspase-8	Heterodimers at DISC active Caspase-8; [20-21]; fitted
33	RIP + TRAF2 + TNFRC2:FLIP:pCasp8	⟶	TNFRC2:FLIP:pCasp8:RIP:TRAF2	0.1 1/s/(µM)^2	---	RIP~TRAF2 recruitment at TNFR Complex2~FLIP~Procaspase-8	RIP and TRAF2 recruitment to p43FLIP [18-19]; fitted
34	TNFRC2:FLIP:pCasp8:RIP:TRAF2 + IKK	⟶	TNFRC2:FLIP:pCasp8:RIP:TRAF2 + IKKa	0.1 1/µM/s	---	IKK activation by TNFR Complex2~FLIP~Procaspase-8~RIP~TRAF2	IKK activation by TNFR Complex2
35	0	⟷	IKK	2e-5 µM/s	0.0001 1/s	IKK turnover	[2]: ka=0.000025; kd=1.25e-4; fitted
36	0	⟷	NFkB	5e-7 µM/s	0.0001 1/s	NF-κB turnover	fitted
37	0	⟷	FLIP	7.03e-7 µM/s	0.0001 1/s	FLIP turnover	fitted ([22]: FLIP overexpression => no death in Type 1)
38	0	⟷	XIAP	0.000241 µM/s	0.0001 1/s	XIAP turnover	[23-24]; fitted
39	0	⟷	A20	3e-6 µM/s	0.0001 1/s	A20 turnover	constitutively produced A20 (versus [2]: 3e-4); fitted
40	IKKa	⟶	0	0.0001 1/s	---	IKK* degradation	[25]
41	IkBa:NFkB	⟶	0	0.0001 1/s	---	IκBα~NF-κB complex degradation	[2, 26]: 2e-5; fitted
42	NFkB_N	⟶	0	3.3e-5 1/s	---	nuclear NF-κB degradation	fitted
43	IkBa_mRNA	⟶	0	0.00013 1/s	---	IκBα-mRNA degradation	http://lgsun.grc.nia.nih.gov/mRNA/index.html, [27]

44	IkBa	⟶	0	0.00154 1/s	---	IκBα degradation	5-10min half life [28]; fitted
45	IkBa_N	⟶	0	3.3e-5 1/s	---	free nuclear IκBα degradation	fitted
46	IkBa:NFkB_N	⟶	0	3.3e-5 1/s	---	nuclear IκBα~NF-κB complex degradation	fitted
47	PIkBa	⟶	0	0.0116 1/s	---	P-IκBa degradation	fitted
48	A20_mRNA	⟶	0	0.00015 5 1/s	---	A20-mRNA degradation	http://lgsun.grc.nia.nih.gov/mRNA/index.html, [27]
49	XIAP_mRNA	⟶	0	3.46e-5 1/s	---	XIAP-mRNA degradation	http://lgsun.grc.nia.nih.gov/mRNA/index.html, [27]
50	FLIP_mRNA	⟶	0	5.47e-5 1/s	---	FLIP-mRNA degradation	http://lgsun.grc.nia.nih.gov/mRNA/index.html, [27]
51	TNFRC1 + IKK	⟶	TNFRC1 + IKKa	300 1/μM/s	---	IKK activation by TNFR Complex1	[29-30]; fitted
52	IKKa	⟶	IKK	0.1 1/s	---	IKK* inactivation	IKK inactivation by autophosphorylation or phosphatases, e.g. by PP2A [25]; fitted
53	TNFRC1 + A20	⟶	TRAF2 + TNF:TNFR:TRADD + A20	0.02 1/μM/s	---	TNFR Complex1 inactivation by A20	RIP at TNFR Complex 1 ubiquitination and proteasomal degradation [31]; fitted
54	NFkB + IkBa	⟶	IkBa:NFkB	4 1/μM/s	---	IκBα NF-κB association	[2, 32]: 0.5; fitted
55	IKKa + IkBa:NFkB	⟶	IKKa + NFkB + PIkBa	0.333 1/μM/s	---	release and degradation of bound IκBα	[33]; fitted
56	NFkB	⟶	NFkB_N	0.0125 1/s	---	NF-κB nuclear translocation	[2] (fitted)

57	NFkB_N	⟶	NFkB_N + IkBa_mRNA	1e-5 1/s	---	IκBα-mRNA transcription	fitted
58	IkBa_mRNA	⟶	IkBa + IkBa_mRNA	0.02 1/s	---	IκBα translation	[2]: 0.5; fitted
59	IkBa	⟷	IkBa_N	0.005 1/s	0.00085 1/s	IκBα nuclear translocation	ka fitted ([2]: 0.001); kd=[2]
60	NFkB_N + IkBa_N	⟶	IkBa:NFkB_N	0.5 1/μM/s	---	IκBα binding NF-κB in nucleus	fitted
61	IkBa:NFkB_N	⟶	IkBa:NFkB	0.005 1/s	---	IκBα_NF-κB N-C export	[2]: 0.01; fitted
62	NFkB_N	⟶	NFkB_N + A20_mRNA	1.25e-5 1/s	---	A20-mRNA transcription	[2, 34]
63	A20_mRNA	⟶	A20 + A20_mRNA	0.005 1/s	---	A20 translation	[2]
64	NFkB_N	⟶	NFkB_N + XIAP_mRNA	1.1e-5 1/s	---	XIAP-mRNA transcription	fitted
65	XIAP_mRNA	⟶	XIAP_mRNA + XIAP	0.0111 1/s	---	XIAP translation	fitted
66	NFkB_N	⟶	NFkB_N + FLIP_mRNA	1.1e-5 1/s	---	FLIP-mRNA transcription	[35]; fitted
67	FLIP_mRNA	⟶	FLIP + FLIP_mRNA	0.00116 1/s	---	FLIP translation	fitted
68	0	⟷	pCasp8	6.17e-5 μM/s	6.17e-5 1/s	Procaspase-8 turnover	fitted
69	0	⟷	pCasp3	1.54e-5 μM/s	6.17e-5 1/s	Procaspase-3 turnover	fitted
70	0	⟷	pCasp6	1.23e-6 μM/s	6.17e-5 1/s	Procaspase-6 turnover	fitted
71	Casp8	⟶	0	5.79e-5 1/s	---	Caspase-8 degradation	fitted

72	Casp3	⟶	0	5.79e-5 1/s	---	Caspase-3 degradation	fitted
73	Casp6	⟶	0	5.79e-5 1/s	---	Caspase-6 degradation	fitted
74	XIAP:Casp3	⟶	0	5.79e-5 1/s	---	XIAP~Caspase-3 complex degradation	fitted
75	0	⟷	BAR	5.13e-7 μM/s	5.79e-6 1/s	BAR turnover	fitted
76	BAR:Casp8	⟶	0	5.79e-5 1/s	---	BAR~Caspase-8 complex degradation	fitted
77	0	⟷	PARP	3.01e-6 μM/s	5.79e-6 1/s	PARP turnover	[3]
78	cPARP	⟶	0	5.79e-6 1/s	---	CPARP degradation	CPARP degradation
79	pCasp3 + Casp8	⟶	Casp8 + Casp3	0.0267 1/μM/s	---	Caspase-3 activation	fitted (same order of magnitude as [3]: 0.06)
80	pCasp6 + Casp3	⟶	Casp3 + Casp6	0.03 1/μM/s	---	Caspase-6 activation	fitted (same order of magnitude as [3]: 0.06)
81	pCasp8 + Casp6	⟶	Casp8 + Casp6	0.000641 1/μM/s	---	Caspase-8 activation	fitted (same order of magnitude as [3]: 0.06)
82	XIAP + Casp3	⟷	XIAP:Casp3	2 1/μM/s	0.001 1/s	XIAP~Caspase-3 complex formation	ka=fitted; kd=[3]
83	XIAP + Casp3	⟶	Casp3	6 1/μM/s	---	XIAP degradation due to Caspase-3	XIAP induced Caspase-3 proteasomal degradation [36]; fitted
84	XIAP:Casp3	⟶	XIAP	5e-5 1/s	---	XIAP~Caspase-3 complex breakup	fitted
85	RIP + Casp3	⟶	Casp3	0.5 1/μM/s	---	negative feedback loop Caspase-3 on TNFR	fitted

						Complex1	
86	FLIP + Casp3	⟶	Casp3	0.5 1/μM/s	---	FLIP degradation by Caspase-3	fitted
87	Casp3 + PARP	⟶	Casp3 + cPARP	0.6 1/μM/s	---	PARP cleavage as Casp3 substrate	[3]
88	BAR + Casp8	⟷	BAR:Casp8	2.08 1/μM/s	0.001 1/s	BAR~Caspase-8 complex formation	ka=fitted; kd=[3]

Table S2.3: List of Compartments

	Name	Volume
1	Cytoplasm	3.2 pl
2	Extracellular	1.34e+3 pl
3	Nucleus	1.06 pl

Mathematical Cell Ensemble Model

Our cytotoxicity experiments consistently revealed that some cells survived TNF treatment, indicating TNF resistance in a subpopulation. Moreover, we observed a broad cell to cell variability in the kinetics of cellular responses. This variance could also be inferred from gradual and partial cytotoxicity responses shown in Figure 2 in the main paper. Reproduction of such heterogeneous population responses requires the identification of the sources of cell variability. Excluding environmental sources, this variability arises from pre-existing differences in the levels of proteins [37]. Indeed, a significant variation in the number of cell surface TNF-R1 receptors across the cell population was observed in our own FACS analysis (Figure S2.1E, blue). The corresponding distribution closely followed a lognormal distribution X, i.e. $X = e^{\mu+\sigma N}$, where N is a normal distribution with zero mean and standard deviation of one. We estimated the parameter σ to 0.148 (see Figure S2.1E, red), and the median e^{μ} was set to 3000 molecules per cell, as obtained from equilibrium binding studies using radio labeled ligand [6]. We assumed all 19 production rates (4 translations via NF-κB, 15 basal productions) followed this distribution around the respective initial values of the nominal cell, independently from each other, and constructed a cell ensemble model, with identical cells except for these varied production rates. The initial condition of each cell was chosen as the steady state with no active caspases.

Experimental Validation of the Cell Ensemble Model

The cell ensemble model was validated with different experiments targeting markers of the NF-κB and the apoptotic module. First, single cell measurements of nuclear NF-κB were obtained using GFP transfection and compared to single cell simulations of the cell ensemble model (Figure S2.2A). Then, average cytoplasmic IκBα concentrations were acquired using Western Blotting (Figure S2.2B-C), and compared to the average IκBα concentration within the cell ensemble model (Figure S2.2D). In both cases, the qualitative behavior of experiments and simulation of the cell ensemble model was similar.

The apoptotic response was quantified using Caspase-3 activity assays (Figure S2.3A), the development of morphological signs of apoptosis in life cell imaging (Figure S2.3B), and cytotoxicity assays (Figure S2.3C-D). Different input signals were applied: continuous or 30 minute pulse stimulation of 3 and 30 ng/ml TNF (Figure S2.3C-D) or microinjection of Caspase-3 (Figure S2.3B). In all of the cases, the experiments and simulations of the proposed cell ensemble model matched nicely.

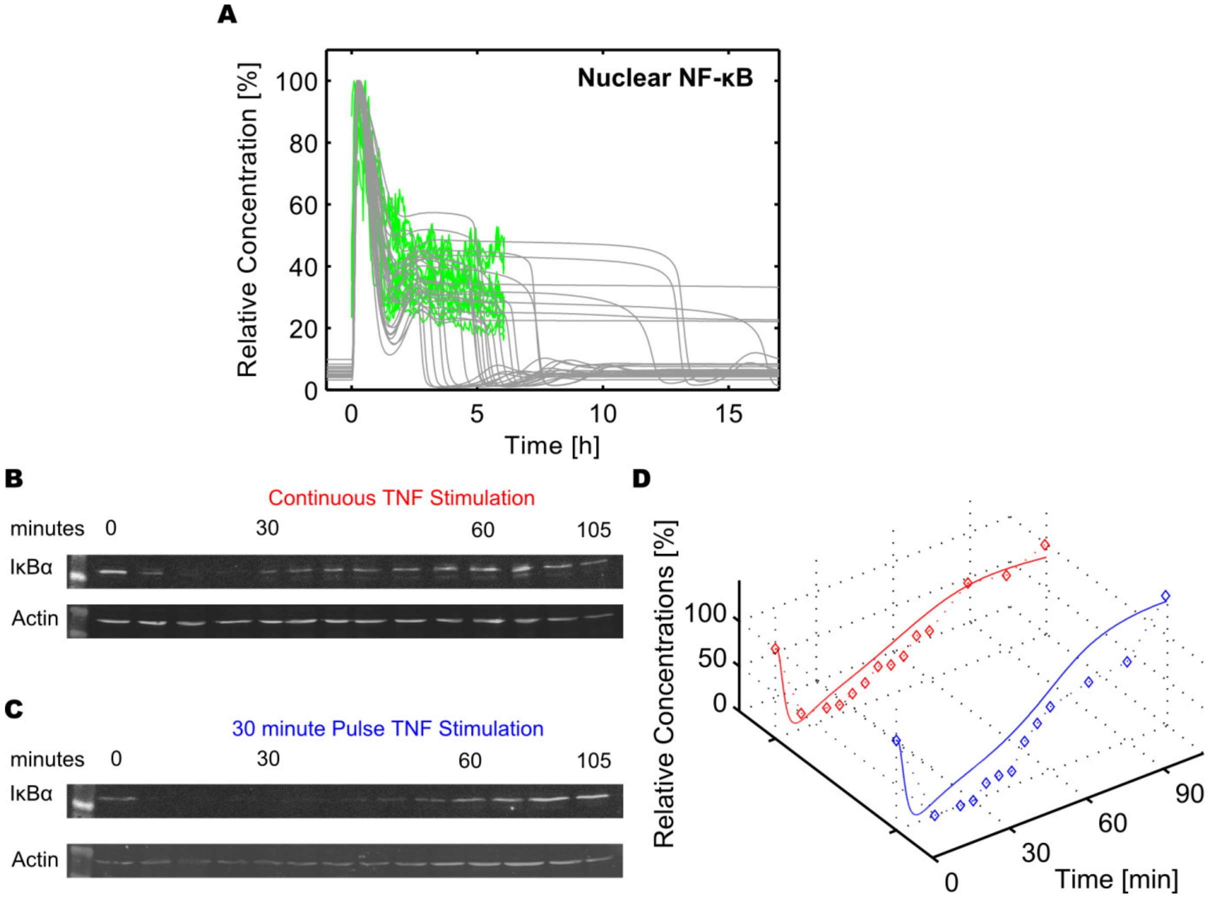

Figure S2.2. Validation of the Cell Ensemble Model by comparison with Experimental Data: NF-κB module. (A) Simulated time courses of NF-κB in the nucleus compared to experimentally obtained fluorescence data from single cells transfected with pEGFP-p65 (green) for 10 ng/ml TNF continuous stimulation. (B)-(C) Experimental data of IκBα (with Actin as loading control) from Western Blotting after 10 ng/ml TNF continuous (B) and 30 minute pulse (C) stimulation over time. (D) Comparison of the experimental data of (B) in red and (C) in blue with the average IκBα concentration as obtained from simulating the cell ensemble model with the corresponding stimuli. Data were normalized by their maximal values.

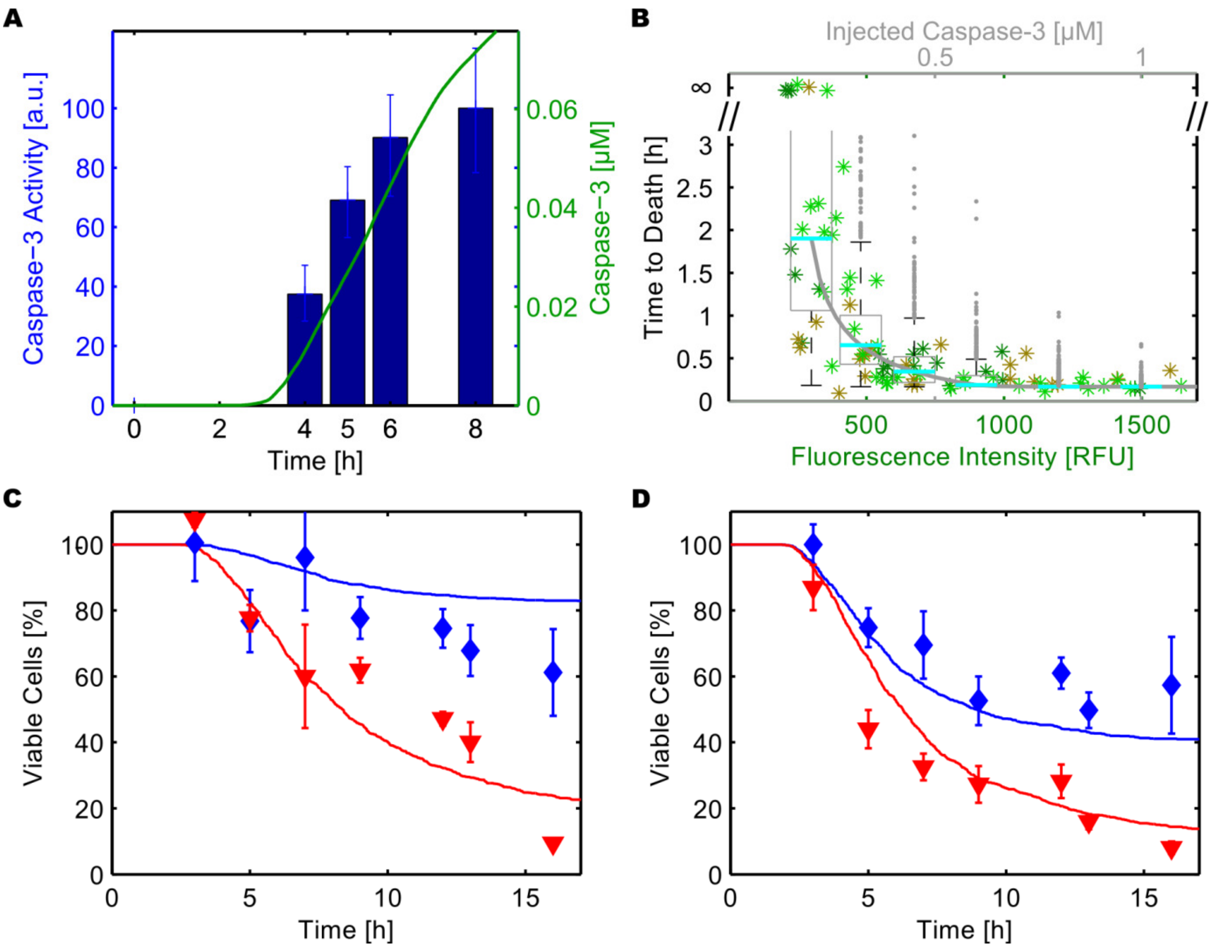

Figure S2.3. Validation of the Cell Ensemble Model by comparison with Experimental Data: Apoptotic Response. (A) Caspase-3 activity assays for continuous stimulation with 1 ng/ml TNF (blue). The error bars show the spread of second and third quartile. In green the corresponding simulation of the cell ensemble model. (B) Time of death as a function of the amount of microinjected activated Caspase-3. Single cells were microinjected with a mixture of Caspase-3 and FITC-Dextran to quantify the relative microinjected amount. For each single cell, a star represents the time to death (development of morphological signs of apoptosis) estimated from time lapse video versus the integrated initial FITC-Dextran fluorescence intensity. The colors differentiate data from three individual experiments. Cells that survived for more than 3 hours were classified as survivors (infinite time to death). In silico microinjection data was obtained by simulating the cell ensemble model 1000 times per stimulus intensity. The solid gray line depicts the median values of the cell populations. For some exemplary stimulus strengths, boxplots show the distributions of the times of death (cyan line: median; light gray box: second and third quartiles; dashed line: whiskers with a maximum length of 1.5 interquartile range; gray dots: outliers). (C) – (D) Experimental cytotoxicity assays (diamonds and triangles; bars indicate standard deviation of three experiments) combined with the respective simulation results. Red: continuous stimulus, blue: 30 minute pulse stimulation. Solid lines: simulation results of the cell ensemble model. (C) 3 ng/ml TNF. (D) 30ng/ml TNF.

Methods

Immunostaining and FACS Analysis

Cell surface TNF-R1 was detected with the TNF-R1-specific antibody H398 (2-4 µg/ml mouse IgG2a, [38]) and a secondary antibody FITC-labeled goat anti-mouse IgG + IgMH+L (Dianova, Hamburg, Germany) at 7 µg/ml. Flow cytometry was performed using a FACSVantage DiVa (Beckmann Coulter Inc, Fullerton, USA) or a Cytomics FC 500 CXP (Beckmann Coulter Inc, Fullerton, USA). Cells were suspended in phosphate-buffered saline (PBS) + 0.05% (v/v) bovine serum albumin (BSA) + 0.02% (v/v) sodium azide (NaN3). As a control, cells were treated with PBA instead of the primary antibody. For each measurement, 10,000 cells were recorded and the data was analyzed using the software CXP analysis 2.2 (Beckmann Coulter Inc, Fullerton, USA).

Preparation of Cytoplasmic Protein Extracts

10^6 KYM-1 cells were seeded in 6-well plates, cultivated over night and stimulated the following day, as mentioned, for each experiment. For intracellular protein quantification via Western Blot analysis, cytoplasmic extracts of KYM-1 cells were prepared as follows. After stimulation, cells were scraped off, washed once with PBS and centrifuged for 5 min at 1,500 rpm. Cytoplasmic and nuclear cell extracts were separately obtained by resuspending pelleted cells in 80 µl WL-buffer (10 mM HEPES pH 7.9, 10 mM NaCl, 0.1 mM EDTA, 5% glycerol, 50 mM NaF, 1 mM DTT), placing them on ice for 15 minutes, adding 1 µl 10% NP-40 and mixing well. Cytoplasmic extracts were obtained as the supernatants resulting from centrifugation at 2,600 rpm for 5 minutes. Total protein concentrations of the supernatants were determined with the Bradford protein assay reagent (Protein Assay, BioRad, Hercules, USA) and photometric extinction measurement at 595 nm. For all samples, identical protein amounts were employed for Western Blots.

SDS-PAGE and Western Blots

Equal amounts of protein were subjected to resolving gels with 12.5 % acrylamide (35 mA for 2 h) and transferred by semi-dry electro blotting (1.5 mA/cm^2 for 90 minutes) onto a nitrocellulose membrane. After blocking with Odyssey Blocking Buffer (Roche Biosciences, Germany; 1x in PBS), incubation with primary antibody of interest was performed in blocking buffer supplemented with 0.2% Tween-20 at 4°C over night. After washing with PBS supplemented with 0.1% Tween-20, samples were incubated with secondary IRdye800-conjugated antibodies in blocking buffer supplemented with 0.2% Tween-20 for 1 hour at room temperature. Immunodetection of protein bands was performed on an Odyssey Infrared Imaging System 2.1.12 (Li-COR Biosciences, Germany). Protein band intensities were quantified using Odyssey software. Rabbit polyclonal antibody directed against IκBα (41 kDa; 1:1,000) was purchased from Cell Signaling Technology®. Polyclonal goat antibody for Actin (43 kDa; 1:1,000) were purchased from Santa Cruz Biotechnology Inc. The secondary IRdye-conjugated antibodies were IRDye 800 CW goat anti-rabbit IgG (LI-COR Bioscience, Lincoln, Nebraska, USA; 1:10,000) and IR Dye 800 CW donkey anti-goat (LI-COR Bioscience, Lincoln, Nebraska, USA; 1:12,000). Prestained Protein Marker, broad range

(6 - 175 kDa) were used. Membranes were stripped in Stripping buffer (LI-COR Bioscience) and then reprobed with the indicated antibodies.

Microinjection

KYM-1 cells for microinjection were grown over night in 35 mm glass-bottomed cell-culture dishes (P35G-1.5-14-C, MatTek, Ashland, MA, USA) in RPMI 1640 supplemented with 5% FCS. Before microinjection, the medium was replaced with phenol red-free Dulbecco's Modified Eagle medium (DMEM) (Invitrogen) supplemented with 5% FCS. Adherent-cell microinjection was performed using pressure injector Femtojet, micromanipulator 5171, microinjection needles Femtotip II, inner diameter ~0.5 µm, and back-loaded using microloaders (all from Eppendorf). The injectate consisted of human, recombinant Caspase-3 (Biovision; 0.42 mg/ml final concentration) with DTT (to keep Caspase-3 in its reduced, active form [39]; 10 mM final concentration) and FITC-Dextran (Molecular Probes; MW 20,000, 2 mg/ml final concentration) and was injected into the cytoplasm of KYM-1 cells (pressure 45 hPa, time 0.2 s). About 50 cells were injected for each experiment. Caspase-3 from different batches was assayed for bioactivity before usage. The increase in fluorescence intensity over time was quantified in a spectrophotometer while letting Caspase-3 digest known amounts of the fluorescent substrate Ac-DEVD-AMC (Alexis Biochemicals; protocol adapted from substrate manufacturer's recommendation). This showed only slight inter-batch variability (data not shown). As a control for microinjection, cells were injected with BSA (0.42 mg/ml final concentration) instead of Caspase-3, the cells survived for at least 8 hours (data not shown). A time-lapse video of the injected cells was acquired (one frame every two minutes, total capture 4-8 h) and cells were scored for integrated average FITC fluorescence intensity (from the first frame that did not contain significant fluorescence contamination due to leaked injectate) and the time to death required to develop morphological signs of apoptosis like blebbing and shrinkage. Time-lapse and analysis was performed in Volocity 4.0.1.

Microscopy

One day before the experiment 3×10^5 cells were seeded in a 35 mm glass bottom microscopy dish (MatTek) and transiently transfected with the respective plasmid. For live cell imaging, the cell culture medium was exchanged with phenol red-free DMEM supplemented with 10% FCS. Cells were placed in the microscope chamber held constantly at 37 °C and 5% CO_2. To induce apoptosis, cells were treated with 10 ng/ml TNF and were observed for the indicated time periods. For control experiments, KYM-1 cells were treated as described above but without stimulation. Live cell imaging experiments, except those using microinjection, were performed with the inverse microscope HS CellObserver (Carl Zeiss). Supplementary pieces of equipment were an AxioCam HR 12 bit camera and a 488nm blue LED illumination by the Colibri system. The object lens was a Plan Apochromat 63x/1.4 oil differential interference contrast (DIC) M27.

For quantitative analyses of microscopic data (except data from microinjection experiments), images were further processed with AxioVision LE 4.7.1 (Carl Zeiss). Regions of interests were defined for signal and background and the fluorescence intensity was tracked over time. Microscope stage for microinjection and subsequent imaging was DM IRB (Leica) with climate controlled at 37°C using Incubator ML, Heating Insert P, FoilCover 35 mm with Cultfoil 25 µm (all PeCon). The light source for fluorescence imaging was Sutter DG4 (Sutter

Instruments), emission and excitation filters and dichroic (Semrock and Chroma). Images and time-lapse video acquired using an ORCA-RT CCD camera (Hamamatsu).

References

1. Eissing T, Conzelmann H, Gilles ED, Allgöwer F, Bullinger E, Scheurich P: **Bistability analyses of a caspase activation model for receptor-induced apoptosis.** *J Biol Chem* 2004, **279:**36892-36897.
2. Lipniacki T, Paszek P, Brasier AR, Luxon B, Kimmel M: **Mathematical model of NF-κB regulatory module.** *J Theor Biol* 2004, **228:**195-215.
3. Albeck JG, Burke JM, Spencer SL, Lauffenburger DA, Sorger PK: **Modeling a snap-action, variable-delay switch controlling extrinsic cell death.** *PLoS Biol* 2008, **6:**2831-2852.
4. Hoffmann A, Levchenko A, Scott ML, Baltimore D: **The IκB-NF-κB signaling module: temporal control and selective gene activation.** *Science* 2002, **298:**1241-1245.
5. Birbach A, Gold P, Binder BR, Hofer E, de Martin R, Schmid JA: **Signaling molecules of the NF-κB pathway shuttle constitutively between cytoplasm and nucleus.** *J Biol Chem* 2002, **277:**10842-10851.
6. Grell M, Wajant H, Zimmermann G, Scheurich P: **The type 1 receptor (CD120a) is the high-affinity receptor for soluble tumor necrosis factor.** *Proc Natl Acad Sci U S A* 1998, **95:**570-575.
7. **Universal Protein Resource (UniProt) database** [http://www.uniprot.org/]
8. **Ensembl human genome database** [http://www.ensembl.org]
9. Boschert V, Krippner-Heidenreich A, Branschadel M, Tepperink J, Aird A, Scheurich P: **Single chain TNF derivatives with individually mutated receptor binding sites reveal differential stoichiometry of ligand receptor complex formation for TNFR1 and TNFR2.** *Cell Signal* 2010, **22:**1088-1096.
10. Bryde S, Grunwald I, Hammer A, Krippner-Heidenreich A, Schiestel T, Brunner H, Tovar GE, Pfizenmaier K, Scheurich P: **Tumor necrosis factor (TNF)-functionalized nanostructured particles for the stimulation of membrane TNF-specific cell responses.** *Bioconjug Chem* 2005, **16:**1459-1467.
11. Krippner-Heidenreich A, Tubing F, Bryde S, Willi S, Zimmermann G, Scheurich P: **Control of receptor-induced signaling complex formation by the kinetics of ligand/receptor interaction.** *J Biol Chem* 2002, **277:**44155-44163.
12. Loetscher H, Stueber D, Banner D, Mackay F, Lesslauer W: **Human tumor necrosis factor alpha (TNF alpha) mutants with exclusive specificity for the 55-kDa or 75-kDa TNF receptors.** *J Biol Chem* 1993, **268:**26350-26357.
13. Van Ostade X, Vandenabeele P, Everaerdt B, Loetscher H, Gentz R, Brockhaus M, Lesslauer W, Tavernier J, Brouckaert P, Fiers W: **Human TNF mutants with selective activity on the p55 receptor.** *Nature* 1993, **361:**266-269.
14. Schoeberl B: **Mathematical modeling of signal transduction pathways in mammalian cells at the example of the EGF induced MAP kinase cascade and TNF receptor crosstalk.** Universität Stuttgart, 2003.
15. Kelliher MA, Grimm S, Ishida Y, Kuo F, Stanger BZ, Leder P: **The death domain kinase RIP mediates the TNF-induced NF-kappaB signal.** *Immunity* 1998, **8:**297-303.

16. Park YC, Ye H, Hsia C, Segal D, Rich RL, Liou HC, Myszka DG, Wu H: **A novel mechanism of TRAF signaling revealed by structural and functional analyses of the TRADD-TRAF2 interaction.** *Cell* 2000, **101:**777-787.
17. Schneider-Brachert W, Tchikov V, Neumeyer J, Jakob M, Winoto-Morbach S, Held-Feindt J, Heinrich M, Merkel O, Ehrenschwender M, Adam D, et al: **Compartmentalization of TNF receptor 1 signaling: internalized TNF receptosomes as death signaling vesicles.** *Immunity* 2004, **21:**415-428.
18. Lamkanfi M, Festjens N, Declercq W, Vanden Berghe T, Vandenabeele P: **Caspases in cell survival, proliferation and differentiation.** *Cell Death Differ* 2007, **14:**44-55.
19. Budd RC, Yeh WC, Tschopp J: **cFLIP regulation of lymphocyte activation and development.** *Nat Rev Immunol* 2006, **6:**196-204.
20. Boatright KM, Deis C, Denault JB, Sutherlin DP, Salvesen GS: **Activation of caspases-8 and -10 by FLIP(L).** *Biochem J* 2004, **382:**651-657.
21. Yu JW, Jeffrey PD, Shi Y: **Mechanism of procaspase-8 activation by c-FLIPL.** *Proc Natl Acad Sci U S A* 2009, **106:**8169-8174.
22. Scaffidi C, Fulda S, Srinivasan A, Friesen C, Li F, Tomaselli KJ, Debatin KM, Krammer PH, Peter ME: **Two CD95 (APO-1/Fas) signaling pathways.** *EMBO J* 1998, **17:**1675-1687.
23. Srinivasula SM, Ashwell JD: **IAPs: what's in a name?** *Mol Cell* 2008, **30:**123-135.
24. Eckelman BP, Salvesen GS: **The human anti-apoptotic proteins cIAP1 and cIAP2 bind but do not inhibit caspases.** *J Biol Chem* 2006, **281:**3254-3260.
25. Delhase M, Hayakawa M, Chen Y, Karin M: **Positive and negative regulation of IkappaB kinase activity through IKKbeta subunit phosphorylation.** *Science* 1999, **284:**309-313.
26. Pando MP, Verma IM: **Signal-dependent and -independent degradation of free and NF-kappa B-bound IkappaBalpha.** *J Biol Chem* 2000, **275:**21278-21286.
27. Sharova LV, Sharov AA, Nedorezov T, Piao Y, Shaik N, Ko MS: **Database for mRNA half-life of 19 977 genes obtained by DNA microarray analysis of pluripotent and differentiating mouse embryonic stem cells.** *DNA Res* 2009, **16:**45-58.
28. O'Dea E, Hoffmann A: **The regulatory logic of the NF-kappaB signaling system.** *Cold Spring Harb Perspect Biol* 2010, **2:**a000216.
29. Mercurio F, Zhu H, Murray BW, Shevchenko A, Bennett BL, Li J, Young DB, Barbosa M, Mann M, Manning A, Rao A: **IKK-1 and IKK-2: cytokine-activated IkappaB kinases essential for NF-kappaB activation.** *Science* 1997, **278:**860-866.
30. Li C, Ge QW, Nakata M, Matsuno H, Miyano S: **Modelling and simulation of signal transductions in an apoptosis pathway by using timed Petri nets.** *J Biosci* 2007, **32:**113-127.
31. Shembade N, Ma A, Harhaj EW: **Inhibition of NF-kappaB signaling by A20 through disruption of ubiquitin enzyme complexes.** *Science* 2010, **327:**1135-1139.
32. Hoffmann A, Levchenko A, Scott ML, Baltimore D: **The IkappaB-NF-kappaB signaling module: temporal control and selective gene activation.** *Science* 2002, **298:**1241-1245.
33. Senftleben U, Cao Y, Xiao G, Greten FR, Krahn G, Bonizzi G, Chen Y, Hu Y, Fong A, Sun SC, Karin M: **Activation by IKKalpha of a second, evolutionary conserved, NF-kappa B signaling pathway.** *Science* 2001, **293:**1495-1499.

34. Krikos A, Laherty CD, Dixit VM: **Transcriptional activation of the tumor necrosis factor alpha-inducible zinc finger protein, A20, is mediated by kappa B elements.** *J Biol Chem* 1992, **267:**17971-17976.
35. Micheau O, Lens S, Gaide O, Alevizopoulos K, Tschopp J: **NF-kappaB signals induce the expression of c-FLIP.** *Mol Cell Biol* 2001, **21:**5299-5305.
36. O'Connor CL, Anguissola S, Huber HJ, Dussmann H, Prehn JH, Rehm M: **Intracellular signaling dynamics during apoptosis execution in the presence or absence of X-linked-inhibitor-of-apoptosis-protein.** *Biochim Biophys Acta* 2008, **1783:**1903-1913.
37. Spencer SL, Gaudet S, Albeck JG, Burke JM, Sorger PK: **Non-genetic origins of cell-to-cell variability in TRAIL-induced apoptosis.** *Nature* 2009, **459:**428-432.
38. Thoma B, Grell M, Pfizenmaier K, Scheurich P: **Identification of a 60-kD tumor necrosis factor (TNF) receptor as the major signal transducing component in TNF responses.** *J Exp Med* 1990, **172:**1019-1023.
39. Salvesen GS, Dixit VM: **Caspase activation: the induced-proximity model.** *Proc Natl Acad Sci U S A* 1999, **96:**10964-10967.
